# Knockdown of Leptin Receptor Affects Macrophage Phenotype in the Tumor Microenvironment Inhibiting Breast Cancer Growth and Progression

**DOI:** 10.3390/cancers12082078

**Published:** 2020-07-27

**Authors:** Luca Gelsomino, Giuseppina Daniela Naimo, Rocco Malivindi, Giuseppina Augimeri, Salvatore Panza, Cinzia Giordano, Ines Barone, Daniela Bonofiglio, Loredana Mauro, Stefania Catalano, Sebastiano Andò

**Affiliations:** 1 Department of Pharmacy, Health and Nutritional Sciences, University of Calabria, 87036 Rende (CS), Italy; luca.gelsomino@unical.it (L.G.); giuseppinadaniela.naimo@unical.it (G.D.N.); rocco.malivindi@unical.it (R.M.); giusy.augimeri@gmail.com (G.A.); salvatore.panza13@gmail.com (S.P.); cinzia.giordano@unical.it (C.G.); ines.barone@unical.it (I.B.); daniela.bonofiglio@unical.it (D.B.); loredana.mauro@unical.it (L.M.); stefania.catalano@unical.it (S.C.); 2 Centro Sanitario, University of Calabria, 87036 Rende (CS), Italy

**Keywords:** breast cancer, leptin, leptin receptor, tumor microenvironment, tumor associated macrophages, immune checkpoint blockade

## Abstract

Aberrant leptin (Ob) signaling, a hallmark of obesity, has been recognized to influence breast cancer (BC) biology within the tumor microenvironment (TME). Here, we evaluated the impact of leptin receptor (ObR) knockdown in affecting BC phenotype and in mediating the interaction between tumor cells and macrophages, the most abundant immune cells within the TME. The stable knockdown of ObR (ObR sh) in ERα-positive and ERα-negative BC cells turned the tumor phenotype into a less aggressive one, as evidenced by in vitro and in vivo models. In xenograft tumors and in co-culture experiments between circulating monocytes and BC cells, the absence of ObR reduced the recruitment of macrophages, and also affected their cytokine mRNA expression profile. This was associated with a decreased expression and secretion of monocyte chemoattractant protein-1 in ObR sh clones. The loss of Ob/ObR signaling modulated the immunosuppressive TME, as shown by a reduced expression of programmed death ligand 1/programmed cell death protein 1/arginase 1. In addition, we observed increased phagocytic activity of macrophages compared to control Sh clones in the presence of ObR sh-derived conditioned medium. Our findings, addressing an innovative role of ObR in modulating immune TME, may open new avenues to improve BC patient health care.

## 1. Introduction

Worldwide, obesity is considered an epidemic health problem as its prevalence is rising both in developed and developing countries [1,2]. This growing incidence has deep clinical implications, since obesity is associated with an expanding set of chronic diseases, such as diabetes, hypertension, cardiovascular, kidney and respiratory diseases and different types of malignancies, including breast cancer [3]. Indeed, it has been widely reported that a complex connection exists between obesity and breast cancer risk and progression sustained by several molecular mechanisms [4,5,6]. Obesity is characterized by an expanded and reprogrammed adipose tissue that is metabolically active. In particular, obesity-associated adipocyte hypertrophy and/or hyperplasia, hypoxia, oxidative stress response, and recruitment of immune cells, such as macrophages, all lead to development of dysfunctional adipose tissue, which produces abundant levels of sex hormones, lipid metabolites, proinflammatory cytokines and adipokines. The same dysfunctional status is displayed by the excess of adipose tissue surrounding breast tissue. In particular, the change in cytokine/adipokine levels is considered to be one of the key features of obesity-related breast cancer [7]. The small polypeptide adipokines are the principal mediators of peritumoral adipose tissue; they locally and systemically act as growth factors in order to promote tumor growth and progression. Among them, the so-called “obesity hormone” leptin, whose circulating levels increase in proportion to fat mass, has been extensively recognized for its role in influencing breast tumor biology [8]. The molecular actions of leptin are mediated through the transmembrane leptin receptor (ObR), encoded by the *db* gene, a member of the class I cytokine receptors [9]. Leptin and its receptor are overexpressed in breast carcinoma especially in higher-grade tumors and are associated with distant metastasis and poor prognosis [10,11,12]. The leptin/ObR axis through the activation of canonical Janus kinase/signal transducers and activators of transcription (JAK2/STATs), the mitogen-activated protein kinase (MAPK/ERK1/2), phosphoinositide 3-kinases/protein kinase B (PI3K/Akt) and non-canonical signaling pathways (Notch, Wnt, mammalian target of rapamycin (mTOR), human epidermal growth factor receptor 2 (HER2/Erb), protein kinase C (PKC), the c-Jun N-terminal kinase (JNK), p38 MAPK, and 5′ AMP-activated protein kinase (AMPK)) [13,14,15] intimately controls breast cancer cell proliferation [13,16,17,18,19,20], migration and invasion, influences epithelial-mesenchymal transition (EMT), supports breast cancer stem cell properties and modulates immune responses [21,22,23,24,25]. With regards to the latter effect, several experimental studies have highlighted the role of leptin in regulating immune cells [20,26], an effect that is involved in indirectly supporting cancer progression. Leptin is also an important mediator for cancer cell–tumor associated macrophage (TAM) interactions. In fact, in vivo studies have demonstrated that adipose tissue within the mammary tumor microenvironment (TME) of obese mice exhibited higher numbers of macrophages and crown-like structures than that of lean tumor-bearers [20,27]. Furthermore, it was shown that leptin stimulates the secretion of interleukins (IL)-8 and -18 by TAMs, thus promoting the malignant phenotype of breast cancer cells [28,29,30]. More recently, we also reported that an enhanced production of leptin from anastrozole-resistant MCF-7 breast cancer cells impacts macrophage behavior within the TME [31].

However, although several studies support the crucial involvement of leptin in breast cancer, definitive conclusions on the biological significance of ObR in breast cancer epithelial cells and on its role in mediating tumor/stroma crosstalk deserve further investigation. Here, we demonstrated, using lentivirus-mediated knockdown strategy, that Ob/ObR signaling integrity in both estrogen receptor (ER)-positive and -negative breast epithelial cancer cells is necessary to maintain the aggressive phenotype of tumor cells and to sustain the pro-tumoral behavior of macrophages in the context of TME.

## 2. Results

### 2.1. siRNA Knockdown of Leptin Receptors Attenuates the Aggressive Phenotype of Human Breast Cancer

To dissect the functional role of ObR in affecting breast cancer phenotype and in sustaining the complex tumor/stroma crosstalk, we stably knocked-down the endogenous expression of ObR in human ER-α positive MCF-7 and in triple-negative MDA-MB-231 breast cancer cells (ObR sh) using lentiviral delivered short hairpin RNA.

**Figure 1:**
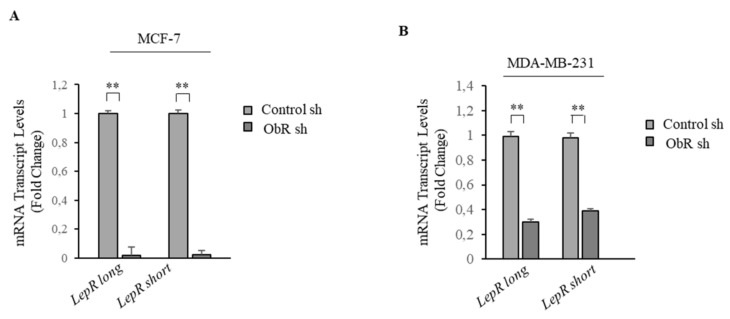
Expression of leptin receptor in ObR sh MCF-7 and MDA-MB-231 breast cancer cells. Real time RT-PCR assay for leptin receptor (long and short isoforms) mRNA expression in control sh and ObR sh MCF-7 (**A**) and MDA-MB-231 (**B**) breast cancer cells. The values represent the mean ± SD of three different experiments, each performed in triplicate. ** *p* < 0.005.

Ob mRNA levels, as measured by quantitative RT-PCR, were significantly down regulated in ObR sh clones as compared to cells stably transfected with a vector shRNA (Control sh) in both cell lines (Figure 1A,B). To confirm that this down regulation had functional consequences in the activity of the cytokine leptin, we performed time course-response studies to analyze phosphorylation of the major leptin downstream effectors. As expected, MCF-7 and MDA-MB-231 ObR sh clones exhibited a marked decrease on the phosphorylation status of JAK2 and STAT3 induced by leptin (Supplementary Figure S1A,B).

Firstly, we used these experimental models to investigate how the integrity of ObR may be essential in preserving the malignant phenotype of breast cancer cells, by evaluating proliferation, migration and invasion abilities of tumor cells.

**Figure 2:**
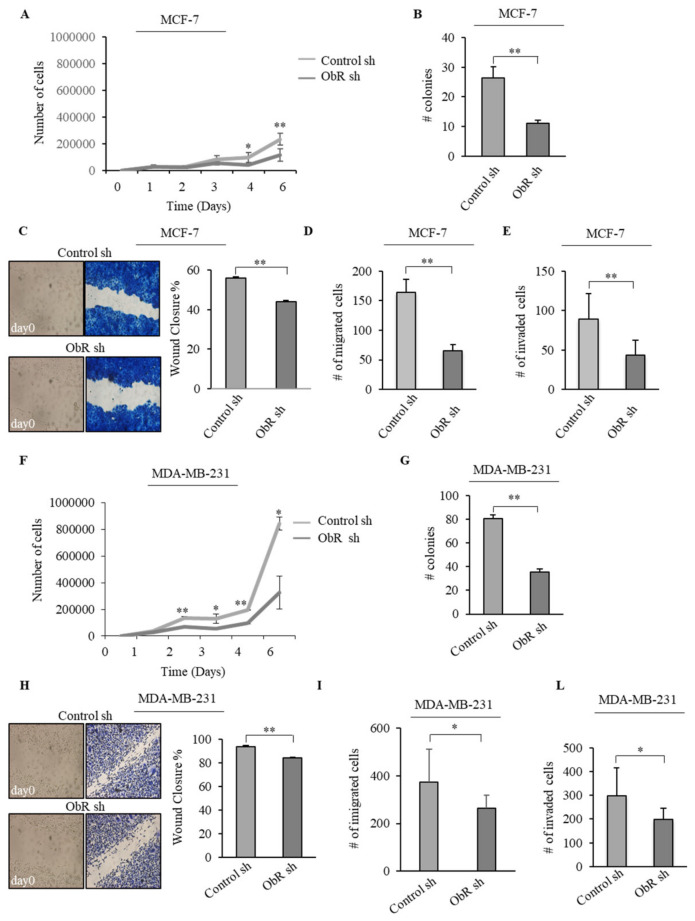
Effects of ObR knockdown on MCF-7 and MDA-MB-231 breast cancer cell phenotype. (**A**,**F**) Growth kinetics and (**B**,**G**) soft agar growth assays of Control sh and ObR sh MCF-7 and MDA-MB-231 breast cancer cells. (**C**,**H**) Wound healing, (**D**,**I**) Boyden chamber transmigration and (**E**,**L**) invasion assays in Control sh and ObR sh MCF-7 and MDA-MB-231 breast cancer cells. Pictures are representative of three independent experiments. The values represent the mean ± SD of three different experiments, each performed in triplicate. * *p* < 0.05, ** *p* < 0.005.

Both MCF-7 and MDA-MB-231 ObR sh clones showed a lower basal proliferative rate when compared to Control sh clones (Figure 2A,F), with a significant reduction in the colony numbers (Figure 2B,G). In addition, MCF-7 and MDA-MB-231 ObR sh clones displayed a reduced cell motility (Figure 2C,D,H,I) and invasion (Figure 2E,L).

**Figure 3:**
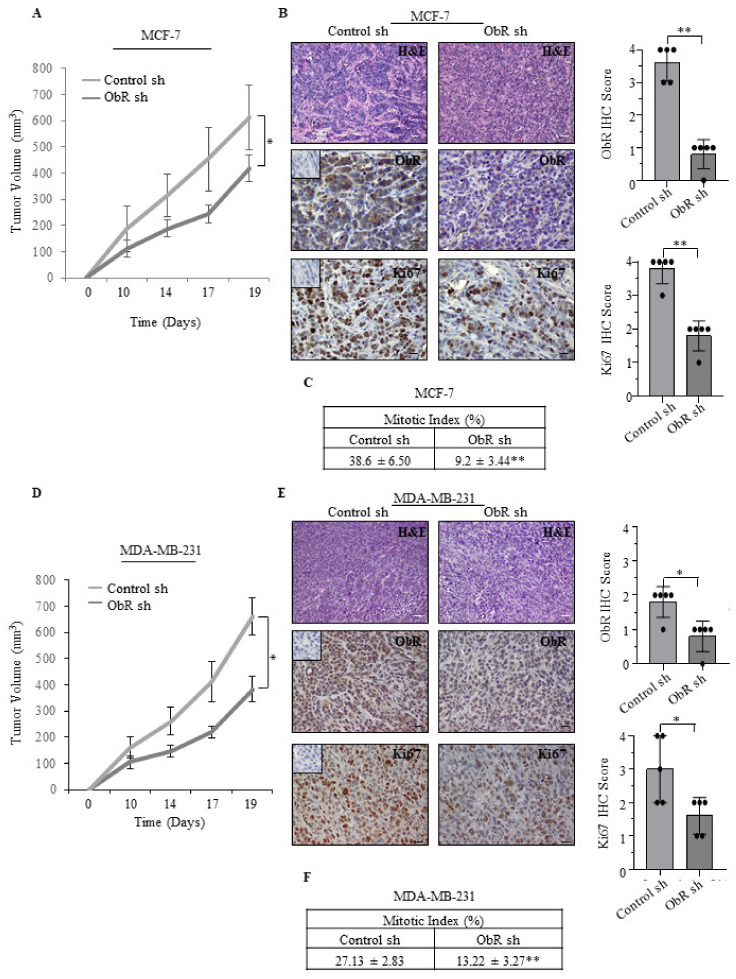
Impact of ObR ablation on tumor growth of MCF-7 and MDA-MB-231 xenografts. (**A**,**D**) Control sh and ObR sh MCF-7 and MDA-MB-231 breast cancer cells were orthotopically injected into nude mice (5 mice/each group). Tumor volume mean ± SD is shown. (**B**,**E**) Representative images of hematoxylin and eosin (H&E), leptin receptor (ObR) and Ki-67 immunohistochemical staining and relative score of Control sh and ObR sh MCF-7 and MDA-MB-231 xenograft tumor sections. Inset, negative control. Scale bar: white = 50 μm, black = 25 μm. (**C**,**F**) Mitotic index in the Control sh and ObR sh MCF-7 and MDA-MB-231 xenograft tumor sections. Values are expressed as mean ± SD. * *p* < 0.05, ** *p* < 0.005.

Furthermore, to evaluate the effects of ObR knockdown on tumorigenesis in vivo, both ObR sh and Control sh MCF-7 and MDA-MB-231 clones were injected into the mammary fat pad of female nude athymic mice and tumor growth was monitored. As shown in Figure 3A,D, a significant reduction in tumor growth of ObR sh clones compared to control ones was observed in both cell lines. These results fit well with a significant decrease in the expression of both Ki67, a well-known marker of cell proliferation (Figure 3B,E and Table 1), and mitotic index (Figure 3C,F) in MCF-7 and MDA-MB-231 ObR sh tumors compared with those revealed in tumors of Control sh mice. 

Therefore, these in vitro and in vivo data provide evidence that knockdown of ObR in breast cancer cells could contribute to attenuation of tumor growth and progression in different cellular backgrounds.

### 2.2. Ob/ObR Signaling Knockdown Hampers Macrophage Recruitment and Influences Their Functional Phenotype in Breast Cancer

Emerging evidence has highlighted an intricate interaction between tumor cells and the surrounding stroma, influencing disease initiation, progression and response to therapy [32]. In the last years, the role of leptin as a mediator of the tumor–stroma interaction has been described [10,33,34,35] but how the lack of its receptor may orchestrate TME is still not completely elucidated. In the tumor stroma, TAMs, the most abundant leukocyte population in mammary tumors, play a critical role in cancer development and progression [36]. Thus, we investigated if ObR knockdown may influence TAM behavior.

**Figure 4:**
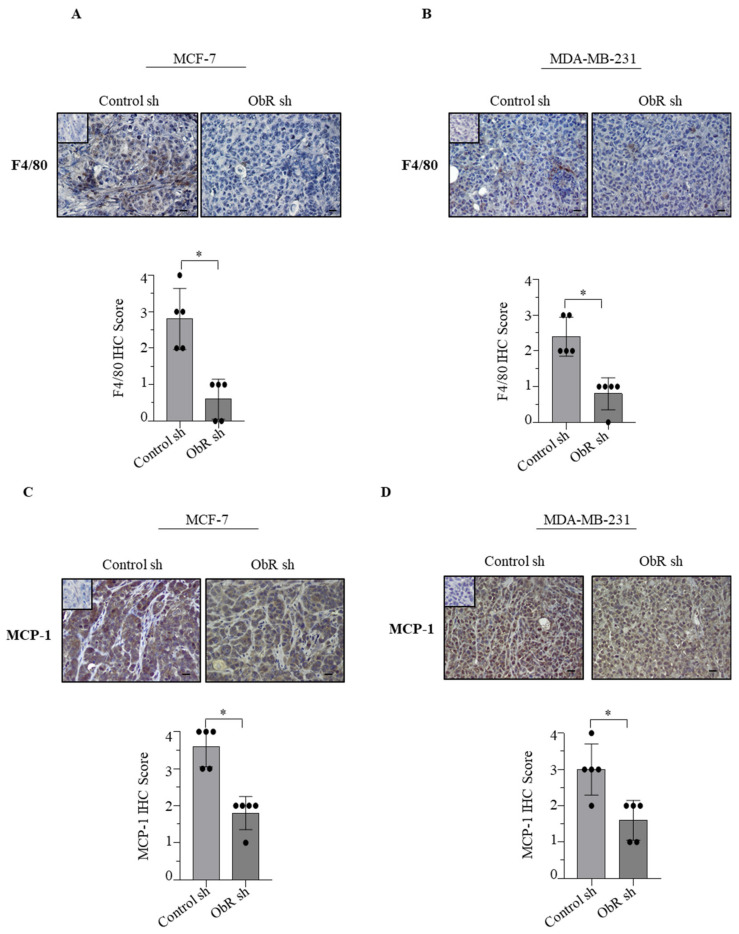
Influence of the lack of ObR on macrophage infiltration and monocyte chemoattractant protein 1 (MCP-1) expression into MCF-7 and MDA-MB-231 xenograft tumors. (**A**,**B**) Immunohistochemical staining and relative score of F4/80 and (**C**,**D**) MCP-1 in Control sh and ObR sh MCF-7 and MDA-MB-231 xenograft tumor sections. Inset, negative control. Scale bar = 25 μm. * *p* < 0.05.

We observed a reduced macrophage infiltration within xenograft tumors from mice injected with transfected ObR sh MCF-7 and MDA-MB-231 cells with respect to the control group as revealed by immunohistochemical staining of F4/80 expression, a unique murine macrophage marker (Figure 4A,B and Table 1). In agreement with these data, decreased expression of monocyte chemoattractant protein-1 (MCP-1), one of the key chemokines, that in tumors facilitates the recruitment and accumulation of TAMs [37], was detected in ObR sh MCF-7 and MDA-MB-231 xenograft tumors compared to Control sh ones (Figure 4C,D and Table 1).

**Figure 5:**
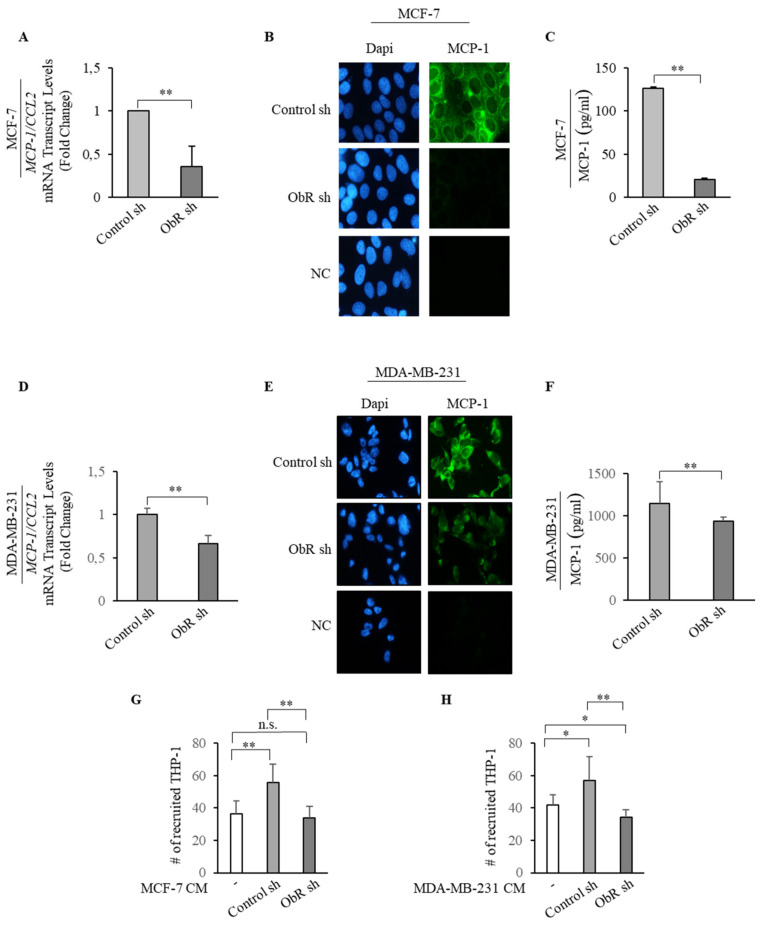
ObR knockdown effects on MCP-1 expression and monocyte recruitment. (**A**,**D**) Real-time RT-PCR assay for *MCP-1*/*C-C Motif Chemokine Ligand 2* (*MCP-1/CCL2*) mRNA expression in Control sh and ObR sh MCF-7 and MDA-MB-231 breast cancer cells. (**B**,**E**) Immunofluorescent staining of MCP-1 protein expression in Control sh and ObR sh MCF-7 and MDA-MB-231 breast cancer cells. DAPI staining was used for nuclei detection (100× magnification). (**C**,**F**) Enzyme linked immunosorbent assay (ELISA) for MCP-1 protein secretion in Control sh and ObR sh MCF-7 and MDA-MB-231 breast cancer cells. (**G**,**H**) Trans-well migration of THP-1 in response to 5% charcoal stripped media (-) and the conditioned medium (CM) derived from Control sh and ObR sh MCF-7 and MDA-MB-231 breast cancer cells was assessed after 5 h incubation. The migrated monocytes were stained with DAPI and six random fields were captured per well with Olympus microscope at 10× magnification. The values represent the mean ± SD of three different experiments, each performed in triplicate. n.s., nonsignificant; * *p* < 0.05; **, *p* < 0.005.

The latter finding well fits with the evidence that in both ObR sh clones, MCP-1 was significantly reduced in terms of mRNA expression (Figure 5A,D), protein content (Figure 5B,E) and amount secreted (Figure 5C,F), addressing how an impairment of Ob/ObR signaling may negatively interfere with MCP-1 expression.

Furthermore, to confirm the reduced recruitment of TAMs observed in ObR sh xenograft tumors, we performed co-culture in vitro experiments that mimic the complex in vivo microenvironment. Thus, THP-1 monocytic cells were incubated with 5% charcoal stripped media, or conditioned medium (CM) collected from ObR sh and Control sh MCF-7 and MDA-MB-231 clones, used as chemo-attractants. CM from Control sh clones increased recruitment of monocytes over basal medium controls; while the migration of monocytes was completely inhibited in the presence of CM derived from ObR sh clones (Figure 5G,H).

**Figure 6:**
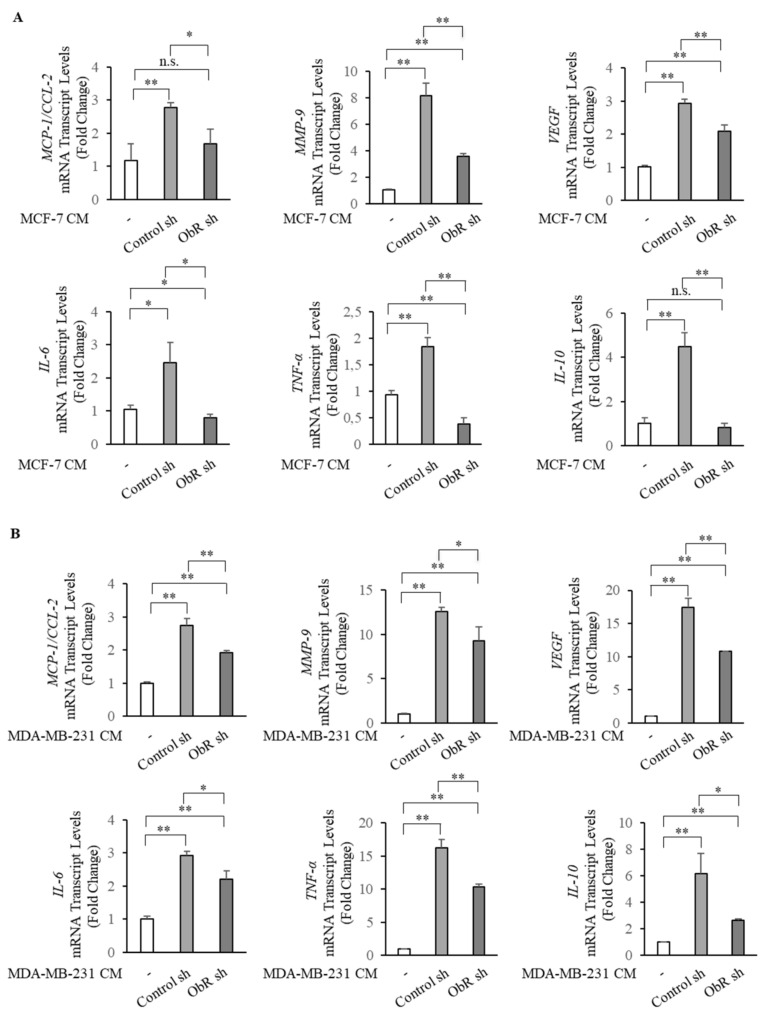
ObR knockdown in breast cancer cells affects the mRNA expression profile of tumor-associated macrophages (TAMs). THP-1 cells were stimulated with phorbol 12-myristate 13-acetate (PMA, 100 nM) for 14 h followed by 24 h rest to obtain THP-1 macrophage-like cells (M0). Real-time RT-PCR assay for *MCP-1/C-C motif chemokine ligand 2* (*MCP-1*/*CCL-2*), *matrix metalloproteinase-9* (*MMP-9*), *tumor necrosis factor alpha* (*TNF-α*), *vascular endothelial growth factor* (*VEGF*), *interleukine-6* (*IL-6*), *inteleukine-10* (*IL-10*) in M0 treated with 5% charcoal stripped media (-) or incubated with conditioned media (CM) derived from Control sh and ObR sh MCF-7 breast cancer cells for 5 days (**A**) and from MDA-MB-231 breast cancer cells for 3 days (**B**). The values represent the mean ±SD of three different experiments, each performed in triplicate. n.s., nonsignificant; * *p* < 0.05; ** *p* < 0.005.

Then, to investigate if ObR knockdown also modulates the phenotype of TAMs, THP-1 macrophage-like cells (M0) were incubated with control media, CM from ObR sh and Control sh clones. As revealed by RT-PCR, we showed increased mRNA levels of several genes usually associated with the TAM phenotype, such as *MCP-1/C-C motif chemokine ligand 2 (MCP-1/CCL-2)*, *matrix metalloproteinase-9 (MMP-9)*, *tumor necrosis factor alpha (TNF-α)*, *vascular endothelial growth factor (VEGF)*, *IL-6*, and *IL-10*, in M0 incubated with CM from Control sh clones with respect to control media (Figure 6A,B). Interestingly a clearly lower induction of these genes was observed in M0 incubated with CM from ObR sh clones compared to Control sh-derived CM (Figure 6A,B).

Taken together, all these data highlight that knockdown of Ob/ObR signaling in breast cancer cells impacts the recruitment of TAMs as well as their functional phenotype.

### 2.3. Leptin Receptor Knockdown Affects the Breast Tumor Immune Microenvironment

TAMs create an immunosuppressive tumor microenvironment that further accelerates tumorigenesis by engaging different mechanisms such as: (i) activation of the immune checkpoint blockade, mainly involving programmed death ligand 1 (PD-L1) and programmed cell death protein 1 (PD-1), (ii) enhanced expression of enzymes hampering immune cell function (i.e., arginase 1) and (iii) by the release of anti-inflammatory cytokines [38,39].

**Figure 7:**
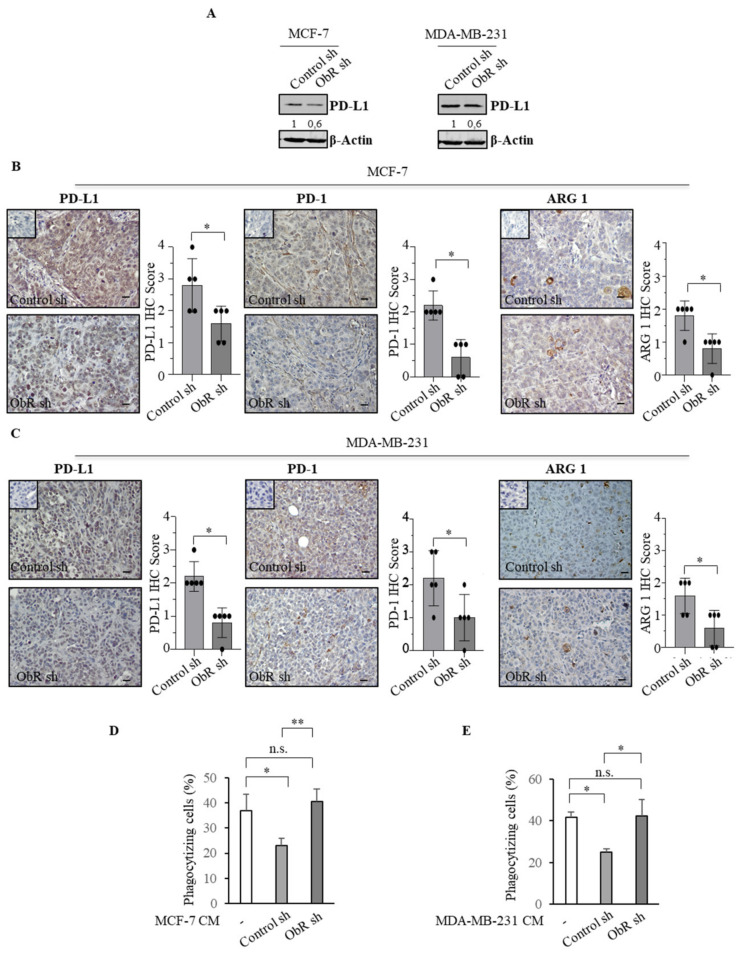
Effects of the lack of ObR on the tumor immune microenvironment. (**A**) Immunoblotting showing programmed death ligand 1 (PD-L1) protein expression in Control sh and ObR sh MCF-7 and MDA-MB-231 breast cancer cells. β-Actin was used as a control for equal loading and transfer. Italicized numbers below blots represent the mean of the band optical density expressed as fold over Control sh ObR cells. PD-L1, programmed cell death protein (PD-1) and arginase (ARG 1) immunohistochemical staining and relative score of Control sh and ObR sh MCF-7 (**B**) and MDA-MB-231 (**C**) xenograft tumor sections. Inset, negative control. Scale bar = 25 μm. THP-1 cells were stimulated with phorbol 12-myristate 13-acetate (PMA, 100 nM) for 14 h followed by 24 h rest to obtain THP-1 macrophage-like cells (M0). Phagocytic activity of M0 treated with 5% charcoal stripped media (-) or incubated with conditioned media (CM) derived from control sh and ObR sh MCF-7 breast cancer cells for 5 days (**D**) and MDA-MB-231 for 3 days (**E**). Cells were incubated with latex beads conjugated with FITC-IgG for 2 h. Pixel intensity of FITC labeled beads was normalized to number of cells and results are expressed as percentage. The values represent the mean ± SD of three different experiments, each performed in triplicate. n.s., nonsignificant; * *p* < 0.05; ** *p* < 0.005.

PD-L1, the primary ligand of PD-1, is expressed in several cancer cells, including those of the breast [40,41]. Thus, we investigated whether the ablation of ObR may also modulate PD-L1 expression in all employed experimental models. Interestingly, we found a decreased PD-L1 expression in both ObR sh clones compared to the control counterpart (Figure 7A). These results were further confirmed in xenograft tumors from mice injected with transfected ObR sh cells compared to Control sh cells (Figure 7B,C and Table 1).

Additionally, in line with the reduced macrophage recruitment previously observed, immunohistochemical analysis of xenograft tumors from mice injected with transfected ObR sh cells revealed a lower expression of PD-1 and arginase 1 (ARG 1) with respect to control ones (Figure 7B,C and Table 1).

The PD-1/PD-L1 axis plays an important role in regulating phagocytosis, the process through which macrophages engulf tumor cells [39]. Thus, we evaluated the phagocytic activity in breast cancer TAMs. As shown in Figure 7D,E, CM-derived from Control sh clones significantly reduced phagocytic capacity of THP-1 macrophage-like cells (M0), while this down-regulation was no longer evident in macrophages treated with CM-derived from ObR sh clones.

These latter results further demonstrated that the integrity of Ob/ObR signaling might be important in modulating the immunosuppressive phenotype of TAMs.

## 3. Discussion

Over the last years, the adipokine leptin has been well recognized as a key member of the molecular network linking obesity to breast cancer. Indeed, hyperactive leptin signaling affects different aspects of breast cancer biology by both modulating the phenotype of neoplastic epithelial cells as well as the behavior of the different components within the TME [13,33,34,42,43]. Nevertheless, how Ob/ObR signaling in breast epithelial cancer cells may impact the behavior of TAMs, one of the major components of the tumor microenvironment [44], still remains to be properly focused.

Unlike tissue-resident macrophages, which are derived largely from the yolk sac in embryogenesis [45], TAMs derive from circulating monocytes. Although in physiological conditions, macrophages control both innate and adaptive immunity through phagocytosis of dead or dying cells and cell debris, tumor cells can reeducate them to a phenotype that deeply supports tumor growth and progression [46,47,48,49,50]. These activities consist of suppression of adaptive immunity by T cells and increased angiogenesis, tumor cell invasion and intravasion into blood vessels. These events are carried out by different subsets of TAMs, which coexist in the tumor stroma in a phenotypic continuum from M1-like (proinflammatory, proimmunity and antitumor macrophages) to M2-like, (antiinflammatory, immunosuppressive, proangiogenic and protumor macrophages) [19]. Moving from these findings, we wondered how the Ob/ObR knockdown signaling in breast epithelial cancer cells could affect tumor capability in shaping its own microenvironment, mainly focusing on its capability to recruit macrophages that can enrich 50% of the entire tumor mass as well as their functional phenotype [44].

In the present study, we demonstrated, using in vitro and in vivo experiments, that in both ER-positive and -negative breast cancer cells the Ob/ObR signaling knockdown turns tumor phenotype into a lesser proliferative and invasive one. In particular, animal studies revealed that both ObR sh MCF-7 and MDA-MB-231 xenografts displayed a markedly reduced growth rate with respect to the control Sh tumors. Interestingly, in ObR sh xenografts we observed a lower content of infiltrate macrophages concomitantly with a lesser expression of MCP-1. These data fit well with our findings obtained in vitro showing how MCP-1 mRNA levels, protein content and secretion were significantly reduced in both ObR sh breast cancer cell lines. The amount of MCP1 secreted by breast tumor cells not only affects the extent of macrophage recruitment but also may influence the cytokine expression featuring the macrophage effector phenotype [35,48,51,52,53,54,55,56]. For instance, the in vitro chemotaxis assay that mimics the microenvironment milieu, showed a reduced capability to recruit human blood monocytes in the presence of the CM-derived from ObR sh clones. In addition, the THP-1 differentiated macrophages incubated in the presence of CM obtained from ObR sh clones, exhibited a decreased expression of cytokines sustaining the multistep development of breast malignancy involved in tumor angiogenesis, invasiveness and metastasis (e.g., MCP-1, VEGF, MMP-9) [57,58,59,60], in EMT and stemness (TNF-α, IL-6) [53,61] and in immunosuppressive effects (IL-10) [62]. Generally, the concentration of macrophage derived IL-10 is almost 10 fold higher than that from leukocytes within the tumor, playing a role in immune suppression in tumors, which is related to tumor drug resistance, cellular growth and proliferation [63]. IL-10 is an important immunosuppressive cytokine fully involved in controlling the expression of another immunosuppressive ligand such as PD-L1. Some findings indicate that in the tumor microenvironment PD-L1 is correlated with the expression level of IL-10. However the relationship between both factors remains unclear [64,65]. It was intriguing to observe how the knockdown of Ob/ObR signaling negatively impacts PD-L1 expression in both ObR sh clones, which fits well with the reduced immunoreactivity of PD-L1 detected in ObR sh tumor xenografts. Previous reports have reported that STAT3 signaling is a regulator of PD-L1 expression in mouse models and cancer cell lines [64]. Moreover, in a large cohort of patients with early breast cancer, a correlation between STAT3 and PD-L1 at both the RNA and protein levels was described [66]. Thus, the reduced expression of PD-L1 could be attributed to the poor activation of STAT3 signaling in ObR sh breast cancer clones. On the other hand, concomitantly with the reduced expression of PD-L1, we observed in both ObR sh xenografts, a decreased immunoreactivity of PD-1, highlighting the involvement of ObR in modulating tumor immune responses.

PD-L1 is widely expressed on different cells, such as hematopoietic cells, including T cells, β cells, macrophages, vascular endothelial cells, epithelial cells, etc., while PD-1 is an inhibitory receptor mainly expressed in activated T cells, β cells, natural killer, dendritic cells and macrophages [67]. PD-1 expression on macrophages increases overtime and with tumor progression [68,69]. When engaged with PD-L1, PD-1 is phosphorylated at tyrosine residues, inducing binding of protein tyrosine phosphatase (PTP1), which can dephosphorylate kinases affecting downstream pathways such as PI3K, Akt, phosphoinositide-specific phospholipase C (PLCγ), extracellular signal-regulated kinases (ERK/RAS). This causes immunosuppression through inhibiting T cell activation, proliferation, survival and cytotoxic function and contributes to cancer progression. Moreover, macrophage-associated PD-1 induces cell apoptosis through negative regulation of Akt activation [18,70,71,72,73]. It was intriguing to observe that the THP-1 differentiated macrophages, in the presence of CM of both ObR sh breast cancer cells showed an increased capability to engulf tumor cells compared to control Sh clones. To explain the latter finding, we may reasonably speculate that the decreased PD-L1 expression in both ObR sh clones may make their CM less agonizing versus PD-1 associated macrophages. Indeed, it has been reported that the lack or a reduced secretion of PD-L1 is associated with an enhanced phagocytic activity of differentiated macrophages [54].

Finally, it is worth mentioning how the expression of ARG 1, as a specific functional marker of TAMs, is reduced in ObR sh xenografts as revealed by immunostaining. TAMs secrete ARG 1 in the microenvironment of different human and mice cancer models [74,75]. ARG 1 metabolizes L-arginine into urea and L-ornithine, depleting it from TME. L-arginine is necessary for T cells function and its depletion inhibits the re-expression of cluster of differentiation 3 (CD3) chains after internalization caused by antigen stimulation and T cell receptor (TCR) signaling. In fact, the expression of ARG 1 is considered the hallmark of M2 macrophage populations [44,63,65].

In conclusion, our findings demonstrate how: (i) the Ob/ObR signaling impacts the biology of breast cancer since its drastic downregulation induces a less aggressive tumor phenotype; (ii) Ob/ObR signaling knockdown contributes to reprograming the recruited macrophages by breast cancer cells by inducing a lower expression of cytokines and immunosuppressive markers concomitantly with their restored capability to engulf tumor cells.

## 4. Materials and Methods

### 4.1. Reagents, Antibodies and Plasmids

Leptin (PHP0013) and puromycin (#A1113803) were acquired from Thermo Fisher Scientific (Waltham, MA, USA). 3-(4,5-Dimethylthiazol-2-yl)-2,5-diphenyltetrazolium bromide (MTT, #M5655) was acquired from Sigma Aldrich (St. Louis, MO, USA). Antibodies were directed to: MCP-1 (#MA5-17040), PD-L1 (#PA5-20343), PD-1 (#MA5-15780) and arginase 1 (#PA5-29645, Thermo Fisher Scientific), ObR (#20966-1-AP, Proteintech Europe, Manchester, UK), β-Actin (#sc-69879, Santa Cruz Biotechnology, Dallas, TX, USA), JAK2 (#3773), pJAK2^Tyr1007/1008^ (#3771), STAT3 (#9132), pSTAT3^Tyr705^ (#9131, Cell Signaling Technology, Denver, MA, USA), Ki67 (#M724029-2, Dako Italia Spa, Milan, Italy) and F4/80 (#ab16911, Abcam, Cambridge, UK).

### 4.2. Cell Cultures

Human MCF-7, MDA-MB-231 breast cancer epithelial cells and the human THP-1 monocytic cell line were from American Type Culture Collection (Manassas, VA, USA). All cell lines were authenticated and stored according to supplier’s instructions. Cells were used within 4 months after recovery of frozen aliquots, and regularly tested for mycoplasma-negativity (MycoAlert Mycoplasma Detection Assay, Lonza, Basilea, CH, Switzerland). To obtain differentiated M0 macrophages, THP-1 cells were seeded in regular growth medium plus phorbol 12-myristate 13-acetate (PMA, 100 nM) for 14 h, followed by 24 h rest to obtain THP-1 macrophage-like cells (M0). Changes in cell morphology related to the monocyte-macrophage transitions were observed and photographed by an Olympus IX-70 microscope.

### 4.3. Lentiviral Transfection

We established stable MCF-7 and MDA-MB-231 cell lines using Control shRNA lentiviral particles-A (#sc-108080) and ObR shRNA (h) lentiviral particles (#sc-36115-V, Santa Cruz Biotechnology) following manufacturer’s instructions. Cells were selected with 1.5 μg/mL (MCF-7) and 3 μg/mL (MDA-MB-231) puromycin overtime to eliminate un-infected cells. *LepR* mRNA expression in stable clones was evaluated by real-time RT-PCR.

### 4.4. Growth Kinetics Assays

Cells were seeded in 12-well plates (10^4^ MCF-7 cells in 1 mL of DMEM medium containing 1% FBS and 5 × 10^3^ MDA-MB-231 cells in 1 mL of DMEM-F12 medium containing 1% FBS) and grown for 1–6 days. The growth rate was determined as previously described [76].

### 4.5. Anchorage-Independent Soft Agar Growth Assays

Anchorage-independent soft agar growth assays were performed as described [77].

### 4.6. Wound Healing Scratch Assays

Wound closure was monitored over 24 h as previously described [78]. Images were acquired at 10x magnification using an OLYMPUS-BX51 microscope. The rate of wound healing was quantified as described [79].

### 4.7. Conditioned Medium Systems

MCF-7 and MDA-MB-231 clones were plated in full media (4 × 10^6^) in 10 cm dish. After 24 h cells were washed twice and cultured with 5% charcoal-stripped serum medium for 24 h. Conditioned medium (CM) was collected, centrifuged, and used in co-culture experiments.

### 4.8. Transmigration/Chemiotaxis Assays

MCF-7 and MDA-MB-231 cells were placed in the upper compartments of Boyden Chamber (8 μm membranes, Corning Costar, Corning, NY, USA) and transmigration assay was performed as described [80].

THP1 cells (10^5^ cells) in 200 µL of phenol-red-free RPMI 1640 were added to the top chamber of a 24-transwell apparatus (5 μm membranes, Corning Costar), while 500 µL of 5% charcoal-stripped serum or of conditioned media derived from breast cells were added to the lower compartment. Cells were incubated for 5 h at 37 °C in an atmosphere containing 5% CO_2_. Migrated cells were fixed and stained with 4′,6-diamidino-2-phenylindole dihydrochloride (DAPI) and quantified by viewing five separate fields per membrane at 20× magnification, using ImageJ (version 1.52t).

### 4.9. Boyden Chamber Invasion Assays

The matrigel-based invasion assay was performed in chambers (8 μm-membranes, Corning) coated with Matrigel (BD Bioscences, 2 mg/mL). Cells under the same experimental conditions as indicated in Section 4.8 were seeded into top transwell-chambers and regular growth medium was used in the lower chambers. After 8–12 h, invaded cells were quantified as reported for transmigration assays.

### 4.10. Real Time RT-PCR Assays

Gene expression was evaluated by real-time reverse transcription (RT)-PCR assessed using SYBR Green Universal PCR Master Mix (Bio-Rad, Hercules, CA, USA). Each sample was normalized on *18S* mRNA content. Relative gene expression levels were calculated as previously described [81]. Primers used are listed in Supplementary Table S1.

### 4.11. Immunofluorescence

Cells were fixed with 4% paraformaldehyde, permeabilized with PBS + 0.2% Triton X-100 followed by blocking with 5% bovine serum albumin for 30 min and incubated overnight with anti-MCP-1 antibody (dilution 1:100) in PBS at 4 °C. The day after the cells were washed three times with PBS and incubated with the secondary antibody anti-mouse IgG-fluorescein isothio-cyanate (dilution 1:200) for 1 h at room temperature. To check the specificity of immunolabeling the primary antibody was replaced by normal mouse serum (negative control). Fluorescence was photographed with an Olympus BX51 microscope (Tokyo, Japan), 100× objective.

### 4.12. Enzyme-Linked Immunosorbent Assay (ELISA)

MCP-1 levels were detected using the ELISA kit (R&D biosystem, Minneapolis, MN, USA) according to the manufacturer’s instructions. Results are presented as pg/mL.

### 4.13. Immunoblot Analysis

Equal amount of protein extracts were subjected to SDS-PAGE as described [82]. Images were acquired using Odissey FC (Licor, Lincoln, NB, USA).

### 4.14. Phagocytosis Assay

THP-1 cells were seeded in 2-well chamber slides, differentiated and incubated with 5% charcoal-stripped serum or of conditioned media derived from breast cells. Macrophages were then assessed for phagocytic activity using the Phagocytosis Assay Kit (Cayman Chemical, Ann Arbor, MI, USA) as previously described [83].

### 4.15. Tumor Xenografts

Female 45-d-old athymic nude mice (Envigo, Milan, Italy) were maintained in a sterile environment and were injected orthotopically with Control sh and shObR MCF-7 and MDA-MB-231 clones (5 × 10^6^). At day 0, estradiol pellets (0.72 mg/pellet, 90-d release; Innovative Research of America, Sarasota, FL, USA) were subcutaneously implanted into the intrascapular region of the mice receiving an inoculation of ERα-positive MCF-7 cells. The next day, cells in 0.1 mL of Matrigel (BD Biosciences, Bedford, MA, USA) were injected orthotopically into the mammary fat pad. Xenograft tumor growth was monitored twice a week by caliper measurements, and tumor volumes (mm^3^) were estimated using the following formula: TV = a × (b^2^)/2, where a and b are tumor length and width, respectively, in millimeters. At day 20, the animals were euthanized following standard protocols. The tumors were dissected from the neighboring connective tissue, frozen in nitrogen, and stored at −80 °C for further analyses [84]. All animals were maintained and handled in accordance with the recommendation of the Guidelines for the Care and Use of Laboratory Animals and experiments were approved by the Animal Care Committee of University of Calabria (OPBA), Italy (ethic code: 533/2019-PR, approved on 19 July 2019).

### 4.16. Histological Analysis

Analysis of tissue sections was performed using tissue fixed in 4% paraformaldehyde for 24 h, dehydrated and embedded in paraffin. Tissue blocks were sectioned at 5 μm and were stained using Mayer’s hematoxylin and eosin to facilitate histology and morphology evaluation. To quantify the mitotic index, the percentage of cells was counted in 10 random fields at 60× magnification (Olympus BX41 microscope).

### 4.17. Immunohistochemistry

For immunohistochemistry, antigen retrieval was performed on 5 μm paraffin sections in 0.01 mol/L citrate buffer (pH 6) in a microwave at low setting. Incubations with primary antibodies were performed at room temperature overnight in a humidified chamber. Primary antibodies used were anti-Ki-67, anti-ObR, anti-F4/80, anti-MCP-1, anti-PD-L1, anti-PD-1 and anti-arginase 1 antibodies. Normal horse or goat serum was used as blocking agent. Biotinylated horse anti-mouse/rabbit (1:100) or biotinylated goat anti-rat (1:100) was used as the secondary antibody and revealed with a Vectastain ABC Kit Elite (Vector Laboratories, Burlingame, CA, USA, PK-6200) and a Peroxidase Substrate Kit DAB (Vector Laboratories, Burlingame, CA, USA, SK-4100). All stained slides were visualized using an Olympus BX41 microscope and the images were taken with CSV1.14 software, using a CAM XC-30 for image acquisition. Immunoreactivity was evaluated by a pathologist in a blinded fashion and scored as: 0, negative; 1, weakly positive; 2, moderately positive; 3, strongly positive; and 4, very strongly positive [79].

### 4.18. Statistical Analysis

Each datum point represents the mean ± SD of at least three independent experiments. In vitro data were analyzed by Student’s *t* test using the Prism 5.0 (GraphPad Software, La Jolla, CA, USA) software program. In vivo data analysis was carried out through a 2-way mixed ANOVA, where the ObR sh was the between-subjects factor. Time over which tumor growth was observed represented the within-subjects factor, which determined repeated measures of tumor volume for the animals used in the experiment.

For immunohistochemistry, the differences in the proportion of positive score between ObR sh and control sh samples were examined by 1-way ANOVA with Bonferroni post hoc testing. A value of *p* < 0.05 was considered statistically significant. Data were analyzed for statistical significance using two-tailed student’s Test, GraphPad-Prism7 (GraphPad Inc., La Jolla, CA, USA). Standard deviations/s.d. are shown.

## 5. Conclusions

Although the negative impact of obesity on breast cancer is widely acknowledged, the optimal treatment for overweight/obese patients is not yet definite and never conceptually referred to the complex interaction existing between breast cancer cells and TME. The novelty of our findings disclose leptin/leptin receptor signaling as one of the main adipose-derived microenvironmental mediators that deeply affects breast tumor biology. Thus, we suggest how the combined therapeutic approaches specifically targeting peritumoral adipose tissue mediators (i.e., Ob/ObR signaling) and removing the immunosuppressed activities of macrophages through the use of neutralizing PD-L1/PD-1 and ARG 1 antibodies may prospectively become extremely promising in the individualized assessed treatment of overweight/obese breast cancer patients.

## Figures and Tables

**Table 1:** Immunohistochemistry scores in MCF-7 and MDA-MB-231 xenograft tumors.

Antibody	MCF-7 Control sh	MCF-7 ObR sh	MDA-MB-231 Control sh	MDA-MB-231 ObR sh
**ObR**	4	1 **	2	1 *
**Ki67**	4	2 **	3	2 *
**F4/80**	3	1 *	2	1 *
**MCP-1**	4	2 *	3	2 *
**PD-L1**	3	2 *	2	1 *
**PD-1**	2	1 *	2	1 *
**ARG 1**	2	1 *	2	1 *

Note: Staining intensity scores are as follows: 0 = negative; 1 = weak; 2 = moderate; 3 = strong; and 4 = very strong. * *p* < 0.05; ** *p* < 0.005 ObR sh clones versus control sh samples. ObR: Leptin Receptor; MCP-1: Monocyte Chemoattractant Protein-1, PD-1: Programmed Cell Death Protein 1; PDL-1: Programmed Death-Ligand 1.

## Supplementary Materials

The following are available online at https://www.mdpi.com/2072-6694/12/8/2078/s1, Figure S1: Leptin signaling in MCF-7 and MDA-MB-231 breast cancer clones; Figure S2: Uncropped Western blots from primary figures are shown; Table S1: Oligonucleotides used in this study.
