# Supplementary material for: Knockdown of Leptin Receptor Affects Macrophage Phenotype in the Tumor Microenvironment Inhibiting Breast Cancer Growth and Progression
Source: Cancers (Basel). 2020 Jul 27;12(8):2078. doi: 10.3390/cancers12082078 (PMC7464041; doi:10.3390/cancers12082078)
Supplement: Supplementary file 1 [file cancers-12-02078-s001.pdf]

cancers

MDPI

Article

Knockdown of Leptin Receptor Affects Macrophage Phenotype in the Tumor Microenvironment Inhibiting Breast Cancer Growth and Progression

Luca Gelsomino, Giuseppina Daniela Naimo, Rocco Malivindi, Giuseppina Augimeri, Salvatore Panza, Cinzia Giordano, Ines Barone, Daniela Bonofiglio, Loredana Mauro, Stefania Catalano and Sebastiano Andò

Supplementary Materials

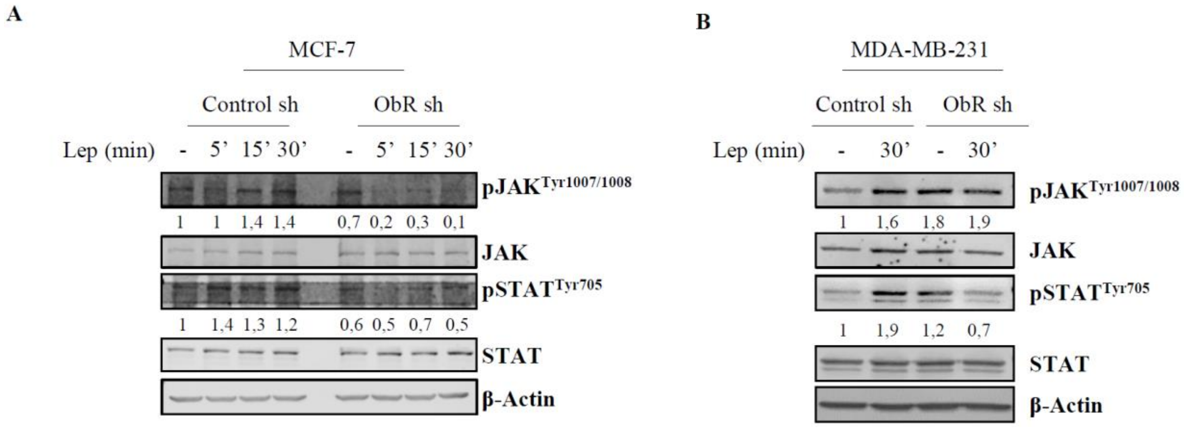

Figure S1. Leptin signaling in MCF-7 and MDA-MB-231 breast cancer clones. Immunoblotting showing phospho JAK$^{Tyr1007/1008}$ and JAK, phospho STAT^{Tyr705} and STAT protein expression in MCF-7 (**A**) and MDA-MB-231 (**B**). β-Actin was used as a control for equal loading and transfer. Italicized numbers below blots represent the mean of the band optical density expressed as fold over Control sh ObR cells. The values represent the mean ± SD of three different experiments, each performed in triplicate.

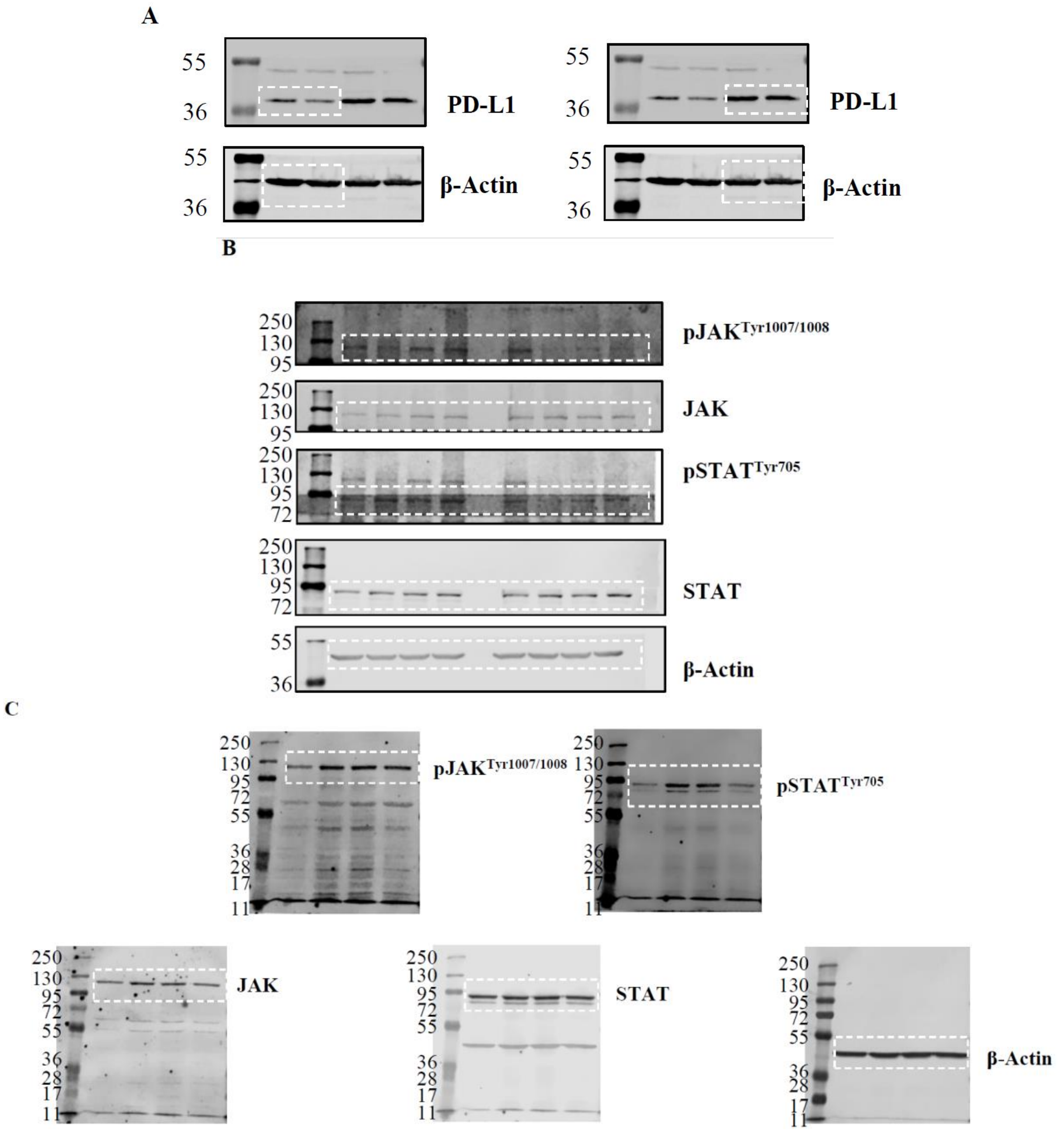

Figure S2. Uncropped western blots from primary figures are shown. (**A**) Figure 7A; (**B**), Supplementary Figure S1A; (**C**) Supplementary Figure S1B.

Table S1. Oligonucleotide primers used in this study.

Gene Name	Gene Symbol	Species		Primer Sequences
Leptin Receptor Long Isoform	*LepR long*	*Human*	Forward<br>Reverse	5′-GATAGAGGCCCAGGCATTTTTTA-3′<br>5′- CACCACTCTCTCTCTTTTTGATTGA-3′
Leptin Receptor Short Isoform	*LepR short*	*Human*	Forward<br>Reverse	5′-ATTGTGCCAGTAATTATTTCCTCTTCC-3′<br>5′-CCACCATATGTTAACTCTCAGAAGTTCAA-3′
18s rRNA	*18s*	*Human*	Forward<br>Reverse	5′-CCCACTCCTCCACCTTTGAC-3′<br>5′-TGTTGCTGTAGCCAAATTCGTT-3′
Monocyte Chemoattractant Protein-1	*MCP-1 /CCL-2*	*Human*	Forward<br>Reverse	5′-CAGCCAGATGCAATCAATGCC-3′<br>5′-TGGAATCCTGAACCCACTTCT-3′
Metalloproteinase 9	*MMP-9*	*Human*	Forward<br>Reverse	5′-AGTTCCCGGAGTGAGTTGAA-3′<br>5′-CTCCACCCTCCCTTTCCTC-3′
Vascular Endothelial Growth Factor	*VEGF*	*Human*	Forward<br>Reverse	5'-GAGATGAGCT TCCTACAGCAC-3′<br>5'-TCACCGCCTCGGCTTGTCACAT-3′
Tumor necrosis factor alpha	*TNF-α*	*Human*	Forward<br>Reverse	5′-GCCCAGGCAGTCAGATCATC-3′<br>5′-GGTTTGCTACAACATGGGCTA-3′
Interleukin 6	*IL-6*	*Human*	Forward<br>Reverse	5′-CCAGGAGCCCAGCTATGAAC-3′<br>5′-CCCAGGGAGAAGGCAACTG-3′
Interleukin 10	*IL-10*	*Human*	Forward<br>Reverse	5′-TCTCCGAGATGCCTTCAGCAGA-3′<br>5′-TCAGACAAGGCTTGGCAACCCA-3′
